# Mucosal Immunity and *Candida albicans* Infection

**DOI:** 10.1155/2011/346307

**Published:** 2011-06-23

**Authors:** David L. Moyes, Julian R. Naglik

**Affiliations:** Department of Oral Immunology, King's College London Dental Institute, King's College London, Floor 28, Tower Wing, London SE1 9RT, UK

## Abstract

Interactions between mucosal surfaces and microbial microbiota are key to host defense, health, and disease. These surfaces are exposed to high numbers of microbes and must be capable of distinguishing between those that are beneficial or avirulent and those that will invade and cause disease. Our understanding of the mechanisms involved in these discriminatory processes has recently begun to expand as new studies bring to light the importance of epithelial cells and novel immune cell subsets such as T_h_17 T cells in these processes. Elucidating how these mechanisms function will improve our understanding of many diverse diseases and improve our ability to treat patients suffering from these conditions. In our voyage to discover these mechanisms, mucosal interactions with opportunistic commensal organisms such as the fungus *Candida albicans* provide insights that are invaluable. Here, we review current knowledge of the interactions between *C. albicans* and epithelial surfaces and how this may shape our understanding of microbial-mucosal interactions.

## 1. Introduction

Fungal diseases became recognised as being of clinical importance in the second half of the last century largely due to a combination of rising numbers of patients with immunodeficiency illnesses such as HIV infections, advances in medical treatments such as cancer therapy and transplantation, and improvements in general life expectancies. The incidence of fungal infections has increased dramatically over the past two to three decades and this trend will inevitably continue into the 21st century, particularly as further improvements are made in health care for immunocompromised patients. Thus, these infections will become an increasingly pressing problem with ever mounting cost pressures on national health facilities.

*Candida* species are the most common fungal pathogens of humans and the causative agents of oral, gastrointestinal, and vaginal candidiasis, giving rise to severe morbidity in millions of individuals worldwide. Vaginal candidiasis alone affects ~75% of women at least once during fertile age [1, 2], equating to ~30 million infection episodes/year. *Candida* infections are also the most common oral manifestation of HIV infection, with 50% of HIV+ patients and 90% AIDS patients suffering from oral candidiasis [3–5]. With ~4 million cases of HIV/year, this equates to ~2 million oral candidiasis cases/year. Indeed, one of the biggest killers of the immunocompromised population is fungal infection. *Candida* species also cause mucosal diseases in the elderly and edentulous individuals, such as *Candida*-associated denture stomatitis. Furthermore, depending on the study, *Candida* infections are also the 3rd or 4th most common hospital-acquired bloodstream infection, making *Candida* species as medically important as many mainstream bacterial infections including Enterococci (*E. coli*) and Pseudomonas spp [6, 7]. In the USA, yearly healthcare costs for systemic fungal infections are ~$2.6 billion, of which *Candida* infections account for ~$1.8 billion [8]. European Union healthcare costs are estimated to be similar. Furthermore, when taking into account mucosal infections, true healthcare costs are likely to be far higher, although precise figures are scarce. Therefore, *Candida* pathogens carry an immense health burden and represent a major socioeconomic challenge for worldwide communities.

Therefore, it is important to understand the mechanisms involved in host-*Candida* interactions, particularly those involved in initiating immune responses and in discriminating between the commensal and pathogenic forms of this fungus. As such, the interactions of *Candida* with cells of the host immune system have been widely studied with several key reports indicating how *Candida* and other fungal species are detected by macrophages, dendritic cells, and neutrophils. However, given that the vast majority of *Candida* infections occur at mucosal surfaces, recent interest has turned towards investigating the interactions between *Candida* and epithelial cells (ECs) and how this might elicit protective immunity. 

## 2. *C. albicans* and Mucosal Surfaces

With the increases in our knowledge of the human microbiome and the interplay with its host, it has become increasingly evident that there exists a highly specialised set of interactions between host organism, residential microbiota, and pathogenic microbes. These interactions lead to either a degree of mutualism in the case of resident, commensal microbes, or breaches in the epithelial barrier followed by disease pathology and immune activation in the case of pathogenic microbes. Exactly how the host discriminates between commensal and pathogenic microbes is not well understood but is key to our understanding of health and disease. Of particular importance is the identification of host mechanisms that discriminate between the commensal and pathogenic states of “opportunistic” microbes, such as the fungus *Candida albicans*, as this will provide valuable insights into how we can manipulate host immunity to control such infections. In particular, understanding these mechanisms will allow us to understand and potentially manipulate immunological events that allow chronic infection by this fungus.

*Candida* species commonly reside as commensal organisms, being part of the normal microbiome in the gut, oral cavity, or vagina in approximately 50% of the population. Although normally these fungi cause no pathology, if there are changes in the local environment, such as alterations in normal microbiota or compromised local immune defences, then these fungi can become pathogenic. As such, they cause mucosal disease in a significant proportion of immunosuppressed patients and women of fertile age [2] with the majority of these individuals experiencing superficial mucosal candidiasis such as thrush. How the host is able to recognise this shift and respond to control these infections is not well understood but has become the subject of growing research interest. Many putative virulence factors have been proposed as playing roles in *C. albicans* infections [9], but of these the most studied and widely accepted is hypha formation leading to invasion [10, 11]. What role, if any, these factors play in host discriminatory responses has, until recently, been unclear, but studies are beginning to show an important role for ECs within mucosal surfaces in this process. Further, the mechanisms that the fungus uses to allow evasion of acute immune responses, resulting in chronic infections, are only now beginning to be understood. In this paper, we will outline current understanding of the interactions of *C. albicans* with mucosal surfaces and discuss the important role ECs play in this process. 

## 3. Immune Recognition of *C. albicans*

The discovery of Toll-like receptors (TLRs) as pattern recognition receptors (PRRs) in the late 1990s lead to a change in our understanding of how pathogens are recognised by the immune system. We now understand that as well as recognition of specific antigens by T-cells (through the T cell receptor) in the adaptive immune response, conserved pathogen associated molecular patterns (PAMPs) are recognised by PRRs as part of the innate immune response. Since the discovery of TLRs, there has been a rapid expansion of characterised PRRs that now includes a panoply of different families and individual molecules. These include the main families of the TLRs, CLRs (C-type lectin receptors), NLRs (Nacht-like receptors), and RLRs (RIG-like receptors) as well as a host of other, individual receptor molecules [12, 13]. Different locations and cell types throughout the body also show differences in the PRRs they express, and, since each PRR recognises individual PAMPs, this may result in differing sensitivities of various body locales to the multitude of microbes that we encounter.

In the case of fungi and particularly *C. albicans*, the predominant immune cell types involved in combating mucosal infection are neutrophils [14]. Recognition of fungal cells by these cells has been the subject of the majority of antifungal immunity research during the last ten years, culminating in the discovery of a new PRR, Dectin-1 (*β*-1,3 glucan) [15, 16], and identification of a role for several other PRRs involved in recognition of different cell wall polysaccharides of this pathogen, including TLR2 (phospholipomannan), TLR4 (O-mannan), and mannose receptor (N-mannan) [17–19] (Table 1). These PRRs have been shown to work both independently and in conjunction with one another. For example, Dectin-1 and TLR2 play a role in the recognition of fungal yeasts, each being responsible for separate actions with Dectin-1 inducing phagocytosis, whilst TLR2 activation induces cytokine production [20, 21]. Each can act independently, but together they produce a synergistic response. Although these are the main receptors used by macrophages and neutrophils, other receptors have also been identified, including Dectin-2 [22], mincle [23], DC-SIGN [24, 25], and galectin-3 [26]. The role of these receptors is currently not fully established and is thus a focus of research by different groups; however, Dectin-2 and DC-SIGN have recently been suggested to play an important role in the recognition of high mannose structures [27] and galectin-3 in the recognition of *β*-1,2 mannosides [26] (Table 1). 

## 4. Epithelial Recognition of *Candida albicans* and Interaction with Immune Cells

Despite our knowledge of the PRR-mediated interactions between myeloid cells and *C. albicans*, the relative importance of these interactions in the detection of this fungus at mucosal surfaces is unclear. This is because, when *C. albicans* colonises a host, there is little evidence that the fungus interacts directly with neutrophils or macrophages in the first instance, rather the initial interaction is with ECs. Therefore, the key question is: do ECs utilise the same PRRs as myeloid cells in the recognition of *C. albicans*? ECs are known to express a range of PRRs such as TLRs, Dectin-1 and galectins along with their coreceptors and adaptors [35–37]. Expression of TLR2 and TLR5, in particular, is expressed at high levels by oral ECs, which is notable given that these receptors have been associated with epithelial growth, survival, and repair [38, 39]. Interestingly, TLR4 is expressed at extremely low levels on oral ECs [35], implying that ECs may be refractory to initial stimulation by TLR4 ligands such as lipopolysaccharide (LPS) and thus Gram-negative bacteria. The exact composition of the PRRs utilised by ECs to recognise *C. albicans* upon initial infection is currently unknown. Recently, we demonstrated that TLR2, TLR4, and Dectin-1 do not appear to be involved in activating epithelial immunity as blockade or inhibition of these receptors did not affect the EC cytokine response to *C. albicans *[40]. This is supported by another study showing a lack of TLR4 involvement in the induction of GM-CSF by *C. glabrata* [41]. Furthermore, although the fungal PAMPs inducing cytokine responses in myeloid cells are well described, including mannans and *β*-glucans, we found that none of these PAMPs or the other polysaccharide constituent of the fungal cell wall, chitin, induced cytokine responses in oral ECs [40]. This was also recently demonstrated for skin keratinocytes [42]. Together, these studies suggest that ECs may utilize different receptors for immune activation and/or target different fungal moieties than myeloid cells, indicating that epithelial fungal detection mechanisms may differ from myeloid cell detection mechanisms.

Despite our knowledge of immune cell- and epithelial-fungal interactions, the relative importance of each interaction in the context of a mucosal infection is unclear. One would, however, expect a high level of immunological cross-talk between the ECs, *Candida,* and local immune cells in order to either maintain homeostasis (commensal state) or to elicit a protective immune response (pathogenic state). To date, this complex but highly interesting area has largely been ignored, but recently it has become evident that these three-way interactions are critical for host defence. One of the ground-breaking studies in this area was undertaken by Weindl et al. [37] using a three-dimensional organotypic oral epithelial model. Such models permit the direct analysis of pathogen-epithelial interactions that are not complicated by nonepithelial factors, and, although these models are not direct mimics of the *in vivo* environment, they can be supplemented with immune cells to investigate more complex cell-cell interactions that are applicable to the *in vivo* situation. Using this model, the authors found that when applied alone, *C. albicans* induced a chemoattractive and proinflammatory cytokine “effector response” but failed to significantly modulate TLR1-10 expression. However, addition of PMNs to the *Candida*-infection model strongly upregulated epithelial TLR4 expression (~100-fold) and protected against *C. albicans* infection. No significant alterations in TLR1-10 expression or protection were observed when PMNs were added in the absence of *C. albicans*. Interestingly, *C. albicans*-induced cell damage was abolished irrespective of whether the PMNs were applied directly to the epithelium or were separated by a membrane. This demonstrated that (i) PMN-dependent protection against *C. albicans* infection was independent of PMN migration or direct cell-cell contact with the oral epithelium, and (ii) three-way communication between *Candida*, EC, and immune cell was essential for TLR4 upregulation and subsequent protection. Further studies demonstrated that *Candida* invasion and cell injury could be restored by TLR4 blockade or “knockdown” of TLR4 using siRNA, even in the presence of PMNs, demonstrating a direct role of epithelial TLR4 in antifungal protective responses. This data is of specific interest as it demonstrates that although epithelial TLR4 is not required for the initial activation of ECs [40], it is required for subsequent epithelial protection in the presence of immune cells. This is the first description of such a PMN-dependent TLR4-mediated protective mechanism at epithelial surfaces and may provide significant insights into how fungal infections are managed and controlled in mucosal tissues. It also demonstrates that far from being bystanders during infections, ECs play an active and integral role in mucosal protection against pathogens. 

## 5. Epithelial Signalling Detection Mechanisms

**Figure 1:**
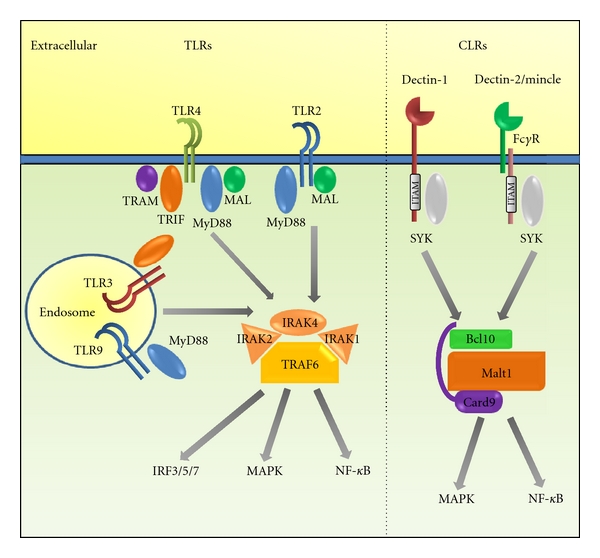
Signal pathway activation by the main TLR and CLR receptors that detect *Candida*. Signalling through TLRs proceeds mainly via TRAF6 with a variety of adaptor proteins acting as intermediaries between receptor and TRAF6. Foremost among these is MyD88 which is utilised by all known TLRs except TLR3. As well as MyD88, there are other adaptor molecules, including TRIF, MAL, and TRAM, with the different TLRs using different combinations of these adaptors. Activation of these adaptors leads to activation of IRAK1, 2, and 4 followed by ubiquitination of TRAF6 which leads to subsequent activation of downstream signalling pathways. Signalling through CLRs utilises cytoplasmic ITAM domains to interact with the SYK adaptor molecule, activating the Card-9-Bcl10-Malt1 protein complex. Some CLRs, such as Dectin-1, include a modified ITAM domain in their cytoplasmic domain. Others, such as Dectin-2, associate with other receptor molecules, notably the FcR*γ* and DAP12 proteins, which possess the ITAM domain that transduces the signal into the cell. In all cases, the net effect is to activate the MAPK and NF-*κ*B pathways, leading to upregulation of specific gene transcription. In addition to this, TLRs are also known to activate transcription via members of the IRF family, including IRF3, IRF5, and IRF7.

Binding of PRRs by their PAMPs is only the first step in the process of recognition. Ligation of PRRs results in activation of a collection of different intracellular signalling pathways which in turn lead to alterations in gene transcription and ultimately changes in protein expression profiles. In myeloid cells, several different signalling pathways are activated by PRR ligation, including the MAPK pathways and the NF-*κ*B pathway (Figure 1). The events leading to the activation of these pathways depend on the triggering receptor. For example, activation of TLRs such as TLR2 and TLR4 leads to interaction of their cytoplasmic TIR (Toll/IL-1 receptor) domain with several different adaptor molecules including MYD88, MAL, TRAM, and TRIF. This results in the assembly of a complex of proteins including IRAK1, 2, and 4 with TRAF6, leading to activation of the MAPK and NF-*κ*B signalling pathways [43], as well as transcription through activation of IRF-3, IRF-5, and IRF-7 [44]. Activation of Dectin-1 and other CLRs leads to activation of similar pathways, although through differing early mechanisms. In contrast to the TLR receptors, CLR ligation results in activation of a CARD9-MALT1-Bcl10 protein complex. This occurs after phosphorylation of an ITAM-like domain in the cytoplasmic domain of the CLR (as with Dectin-1) or co-receptor (e.g., FcR*γ* for Dectin-2) results in phosphorylation of SYK [15, 45]. Subsequent to these events is the activation of both NF-*κ*B and MAPK signalling and downstream transcriptional events such as activation of the MAPK transcription factor AP-1 heterodimer. Activation of the MAPK pathway also leads to activation of a group of phosphatases known as the dual specificity phosphatases (DUSPs) including MKP1. These phosphatases act to dephosphorylate and thus deactivate the MAPK proteins, ERK1/2, p38, and JNK [46]. As these phosphatases are activated by the MAPK proteins as a result of MAPK signalling, they form part of a negative feedback loop to regulate the activity of these pathways with each DUSP being specific for a different MAPK protein.

The exact roles of these pathways in normal, healthy responses to *Candida* vary depending on the celltype and the context of the stimulation. Different cell types express varying levels of the relevant transcription factors, resulting in alternate transcriptional profiles being activated. Equally, the combination of PRRs on the surface of different cells will result in alternate mechanisms being activated. Currently, almost all studies investigating cellular responses to *Candida* infection have been carried out using myeloid or lymphoid cells. However, given that most pathogens effect through a mucosal surface, detailed analysis of the responses of other cell types that comprise these surfaces, particularly ECs, may identify novel and unusual mechanisms for host-microbe interactions. Such studies may also identify mechanisms that enable a host to distinguish between commensal and pathogenic microbes in general or between the commensal and pathogenic state of individual organisms, including *C. albicans.*

**Figure 2:**
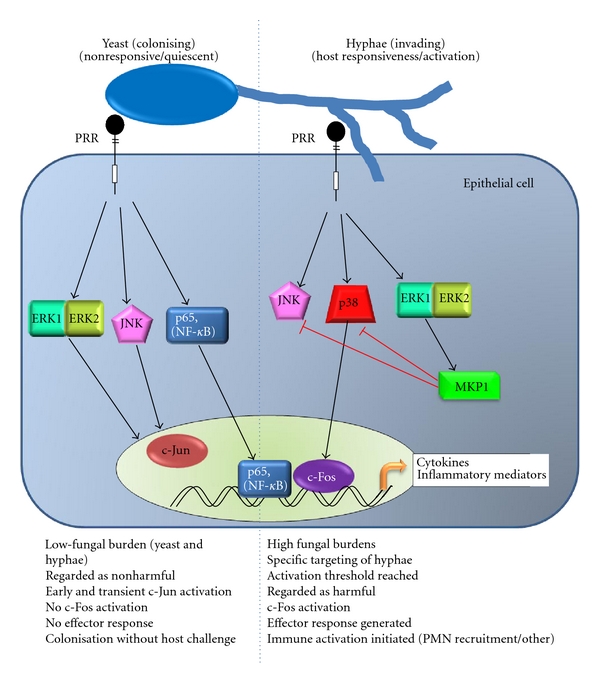
Epithelial cell recognition of *C. albicans*. Epithelial cells recognise *C. albicans* via a two-step process [40]. Initial recognition of yeast by surface PRRs results in prolonged activation of NF-kB and an early transient activation of MAPK signalling leading to activation of c-Jun through the ERK1/2 and JNK pathways. When the burden of hyphae passes a threshold, recognition of these hyphae triggers a second, prolonged activation of MAPK signalling. This results in activation of MKP1 through the ERK1/2 pathway and c-Fos via p38 signalling. NF-*κ*B and c-Fos then play essential roles in the transcription of cytokines secreted by the epithelial cells, whilst MKP1 acts as a negative regulator to control the activation of JNK and p38 signalling.

Several previous investigations have identified NF-*κ*B and MAPK signalling as the main response mechanisms to bacterial infection in ECs [47, 48]. Further studies have identified NF-*κ*B as an important pathway in EC responses to *Candida* infections [18, 49]. In our own recent study, we analysed the responses of oral ECs over time to *Candida* infection and identified a unique mechanism that enables these cells to discriminate between the yeast and hyphal form of *C. albicans*, which we believe has strong correlations as to whether the fungus is viewed by the host as “pathogenic” or “commensal” [40]. We confirmed that the NF-*κ*B pathway is important in oral EC responses to this fungus. However, we identified MAPK signalling through all three pathways as the mechanism by which oral ECs identify when this fungus becomes invasive and pathogenic. Whilst NF-*κ*B signalling increases linearly over time, MAPK activation shows a bi-phasic response in which a transient, early response through ERK1/2 and JNK signalling induces c-Jun activity in response to the presence of *Candida* (yeast or hyphae). A second prolonged, late response induced in response to hyphae drives further ERK1/2 and p38 signalling, activating MAPK regulation via the MAPK phosphatase MKP1 and induces c-Fos activity resulting in production of cytokines. Of particular importance is that this second MAPK response is only induced when a sufficient fungal hyphal burden is present, demonstrating that a threshold level of activation needs to be reached prior to epithelial immune activation (Figure 2). This MAPK-based discriminatory pathway may, therefore, provide a mechanism for epithelial tissues to remain quiescent in the presence of low-fungal burdens whilst responding specifically and strongly to hyphae when burdens increase. We propose that this mechanism may comprise a “danger response” pathway, which may be critical in identifying when this normally commensal fungus has become pathogenic. Failure or deficiencies in this “danger response” mechanism could lead to a potentially chronic infection that is inefficiently managed by the host, as no “danger” signal would be elicited to recruit an immune response. Thus, it appears that ECs are instrumental in discriminating between the commensal and pathogenic states of opportunistic pathogens and that this discrimination is communicated via the MAPK pathway. 

## 6. *Candida*-Induced EC Cytokine Responses

Recognition of *Candida* by host cells leads to activation of a cytokine response profile. For myeloid cells, this profile is fairly well documented and includes release of IL-12, IL-1*α*/*β* and TNF*α* along with other proinflammatory cytokines [17]. Although less well defined, the EC effector response has to some extent been described. We and others have shown that infected ECs produce cytokines and chemokines with a proinflammatory profile [37, 40, 50–52]. Among these are included IL-1*α*/*β*, IL-6, G-CSF, GM-CSF, and TNF*α* as well as the chemokines RANTES, IL-8, and CCL20*.* In contrast to myeloid and lymphoid cells, however, ECs do not produce IL-12, IFN*γ*, IL-4 or IL-13. What direct effect these cytokines have on epithelial protection is unclear, although, given that these cytokines act upon both lymphoid and myeloid cells it is likely that they are involved in activating and recruiting these cells into the mucosal layer. For example, IL-8 will recruit neutrophils to the epithelium, subsequently inducing neutrophil-dependent mucosal defence against *C. albicans* [37]. 

As well as cytokines and chemokines, *Candida* infection of ECs results in an increase in expression of matrix metalloproteases (MMPs) [53] which will play a role in remodelling of the epithelium and modulating the barrier function. Infection also results in the upregulation of various antimicrobial peptides such as *β*-defensins and LL-37 [54, 55]. These antimicrobial peptides have anticandidacidal activity and play a significant role in combating infections and invasion as well as initiating other immune responses [56, 57]. 

## 7. Mucosal Surface Immune Responses to *C. albicans*

 The secretion of cytokines and chemokines by ECs in response to *Candida* invasion will result in the recruitment, differentiation, and activation of a variety of immune cells, including neutrophils, dendritic cells and T cells. The role of neutrophils in anti-*Candida* mucosal immunity appears to be twofold. As described earlier, neutrophils can induce EC-mediated protection against *C. albicans* infections through upregulation of TLR4 [37]. Neutrophils can also directly kill *Candida* cells through ingestion and killing, degranulation, or through the recently discovered Neutrophils extracellular Traps (NETs). NETs occur as a specialised form of neutrophils cell death and comprise a web of chromatin “fibres” coated with serine proteases, antimicrobial proteins, and other neutrophils contents which capture and kill *C. albicans* on various surfaces [58, 59].

As well as IL-8-recruited neutrophils, secreted CCL20 will recruit the Th17 T cell subset [60]. These cells secrete IL-17 and IL-22 and have been associated with anti-*Candida* immunity [61]. Recently, dendritic cell recognition of fungi through Dectin-1 and Dectin-2 has been shown to play an instrumental role in driving development of Th17 cells [62], indicating a relationship between fungal infections and this important T cell subset. The exact role of these cells in anti-*Candida* immunity is not fully understood, with evidence to suggest both a positive [63, 64] and negative [65] role, although it is becoming clear that cytokines secreted by these cells, most notably IL-17 [66–68] and IL-22 [69], play a significant role in antifungal immunity. IL-17 acts on ECs and neutrophils, functioning as a bridge between the adaptive and innate immune responses. Its effects on ECs include induction of antimicrobial peptides, MMPs, and other inflammatory mediators. The role of L-17 in anti-*Candida* immunity is controversial with evidence to indicate that it both increases [65] and reduces [67, 68] *C. albicans* burdens after infection through various routes. Interestingly, infection of peripheral blood mononuclear cells by live *C. albicans* results in inhibition of IL-17 secretion resulting from the effects of *C. albicans*-released 5-hydroxytryptophan metabolites [70] with a concurrent suppression of immunity to the fungus, suggesting that IL-17 may be important in coordinating immune responses to the fungus. IL-22 has similar effects to IL-17 on ECs but has been suggested to control yeast cell growth, as well as controlling epithelial layer integrity during infection [69], thus helping to control cell numbers and invasion of the epithelium during an infection event. The importance of the Th17 response in mucosal immunity to *Candida* spp infections is underlined by several recent studies linking defects in the Th17 response and production of IL-17 to cases of chronic mucocutaneous candidiasis (CMC) [67, 71]. This link is further supported by the finding that in cases of autoimmunity with neutralising antibodies to Th17 cytokines (IL-17A, IL-17F, and IL-22), there is an increased incidence of CMC [72]. Despite this, the report that IL-17 may play a deleterious role in anti-*Candida* immunity [65] suggests that the situation may be far more complex *in vivo*.

As well as driving innate immunity and neutrophil responses, T_h_17 cells have also been shown to drive antibody responses at mucosal surfaces, in particular secretory IgA (sIgA). In mice, Th17 cells induce an influx of CD19^+^ B cells and boost levels of sIgA as well as epithelial expression of polymeric IgA receptor [73]. Increases in secreted IgA at mucosal surfaces have previously been reported [74], indicating that this may be another mechanism by which T_h_17 responses mediate protection against mucosal candidiasis, especially since sIgA antibodies can inhibit the adherence of *C. albicans* to epithelial cells [75]. 

## 8. Conclusions

Until recently, our understanding of the events and mechanisms involved in host mucosal responses to fungal cells was elementary. In particular, the role of ECs in these events was considered to be relatively unimportant. Recent advances in our knowledge of immunity have resulted in the identification of a novel subset of T cells that produce cytokines targeting ECs, thus forming a direct link involved in maintaining the mucosal barrier. Equally, the discovery of an EC-driven mechanism for protection against *Candida* infection demonstrates that these cells communicate with immune cells and play a role in combating fungal infections. The elucidation of an epithelial-specific mechanism for identifying the pathogenic state of *C. albicans *has confirmed the importance of ECs in mediating protective mucosal mechanisms and in discriminating commensal microbes from pathogens. The identification of this role for ECs may open new avenues of research for treatments for use in immunocompromised patients or for those with chronic mucosal infections. 

## Figures and Tables

**Table 1:** Pattern recognition receptors that sense fungal-associated PAMPs.

Family	Receptor	PAMP	References
TLRs	TLR2	Phospholipomannan	[19]
	TLR3	Double-stranded RNA	[28]
	TLR4	Mannan	[29]
		O-linked Mannan residues	[21]
	TLR9	CpG DNA	[30]

CLRs	Dectin-1	*β*-1,3-glucan	[16]
	Dectin-2	High-mannose structures	[31]
		*α*-mannans	[22]
	Mannose receptor	Mannan	[32]
	MINCLE	Unknown	[23]
	Galectin-3	*β*-1,2-Mannosides	[26]
	DC-SIGN	High-mannose structures	[24]

NLRs	NLRP3	Unknown	[33]

Others	Cdw17	Unknown	[34]
